# Impact of High-Cut-Off Dialysis on Renal Recovery in Dialysis-Dependent Multiple Myeloma Patients: Results from a Case-Control Study

**DOI:** 10.1371/journal.pone.0154993

**Published:** 2016-05-06

**Authors:** Hans U. Gerth, Michele Pohlen, Dennis Görlich, Gerold Thölking, Martin Kropff, Wolfgang E. Berdel, Hermann Pavenstädt, Marcus Brand, Philipp Kümpers

**Affiliations:** 1 Department of Medicine D, Division of General Internal Medicine, Nephrology, and Rheumatology, University Hospital of Muenster, Muenster, Germany; 2 Department of Medicine A, Hematology and Oncology, University Hospital of Muenster, Muenster, Germany; 3 Institute of Biostatistics and Clinical Research, University Hospital of Muenster, Muenster, Germany; Bambino Gesù Children's Hospital, ITALY

## Abstract

**Background:**

High-cut-off hemodialysis (HCO-HD) can effectively reduce high concentrations of circulating serum free light chains (sFLC) in patients with dialysis-dependent acute kidney injury (AKI) due to multiple myeloma (MM). Therefore, the aim of this study was to analyze renal recovery in a retrospective single-center cohort of dialysis-dependent MM patients treated with either conventional HD (conv. HD) or HCO-HD.

**Methods and Results:**

The final cohort consisted of 59 patients treated with HCO-HD (n = 42) or conv. HD (n = 17). A sustained sFLC response was detected in a significantly higher proportion of HCO-HD patients (83.3%) compared with conv. HD patients (29.4%; p = 0.007). The median duration of sFLC required to reach values <1000 mg/l was 14.5 days in the HCO-HD group and 36 days in the conv. HD group. The corresponding rates of renal recovery were 64.3% and 29.4%, respectively (chi-squared test, p = 0.014). Multivariate regression and decision tree analysis (recursive partitioning) revealed HCO-HD (adjusted odds ratio [OR] 6.1 [95% confidence interval (CI) 1.5–24.5], p = 0.011) and low initial uric acid values (adjusted OR 1.3 [95%CI 1.0–1.7], p = 0.045) as independent and paramount variables associated with a favorable renal outcome.

**Conclusions:**

In summary, the results from this retrospective case-control study suggest in addition to novel agent-based chemotherapy a benefit of HCO-HD in sFLC removal and renal outcome in dialysis-dependent AKI secondary to MM. This finding was especially pertinent in patients with low initial uric acid values, resulting in a promising renal recovery rate of 71.9%. Further prospective studies are warranted.

## Introduction

Several improvements in the treatment of multiple myeloma (MM) have emerged in the past few decades, yielding improved responses and overall survival. Novel therapeutic agents can even reverse moderate renal impairment (RI) related to MM,[1] but the incidence of dialysis-dependent end-stage renal disease has not declined significantly over time.[2] In particular, dialysis-dependent patients still have a poor prognosis.[2–5] In this regard, recovery from acute kidney injury (AKI) caused by MM is even more predictive of survival than response to chemotherapy.[4,6]

In addition to reversible factors such as hypercalcemia, the most common cause of AKI in MM patients is a tubulointerstitial pathology that results from the very high circulating concentrations of monoclonal immunoglobulin free light chains. These endogenous proteins can result in isolated proximal tubule cell cytotoxicity, tubulointerstitial nephritis, and cast nephropathy.[7,8] AKI may become irreversible if serum free light chain (sFLC) concentrations are not rapidly reduced. In addition to the application of chemotherapeutic agents, the reduction of high sFLC concentrations can be achieved by extracorporeal techniques.

High-cut-off hemodialysis (HCO-HD) using special filters with a sieving coefficient up to a molecular weight of 45 kDa has been established as an effective procedure to remove sFLC in MM patients.[9–12] Approximately three-quarters of patients receiving chemotherapy plus HCO-HD reached dialysis independency.[10–12] However, neither retrospective nor prospective data on the additional benefit of HCO-HD in combination with modern systemic therapy have been published to date. Therefore, the aim of this study was to analyze renal recovery in a retrospective single-center cohort of dialysis-dependent MM patients treated with either conventional HD (conv. HD) or HCO-HD.

## Methods

### Patients and study design

**Fig 1:**
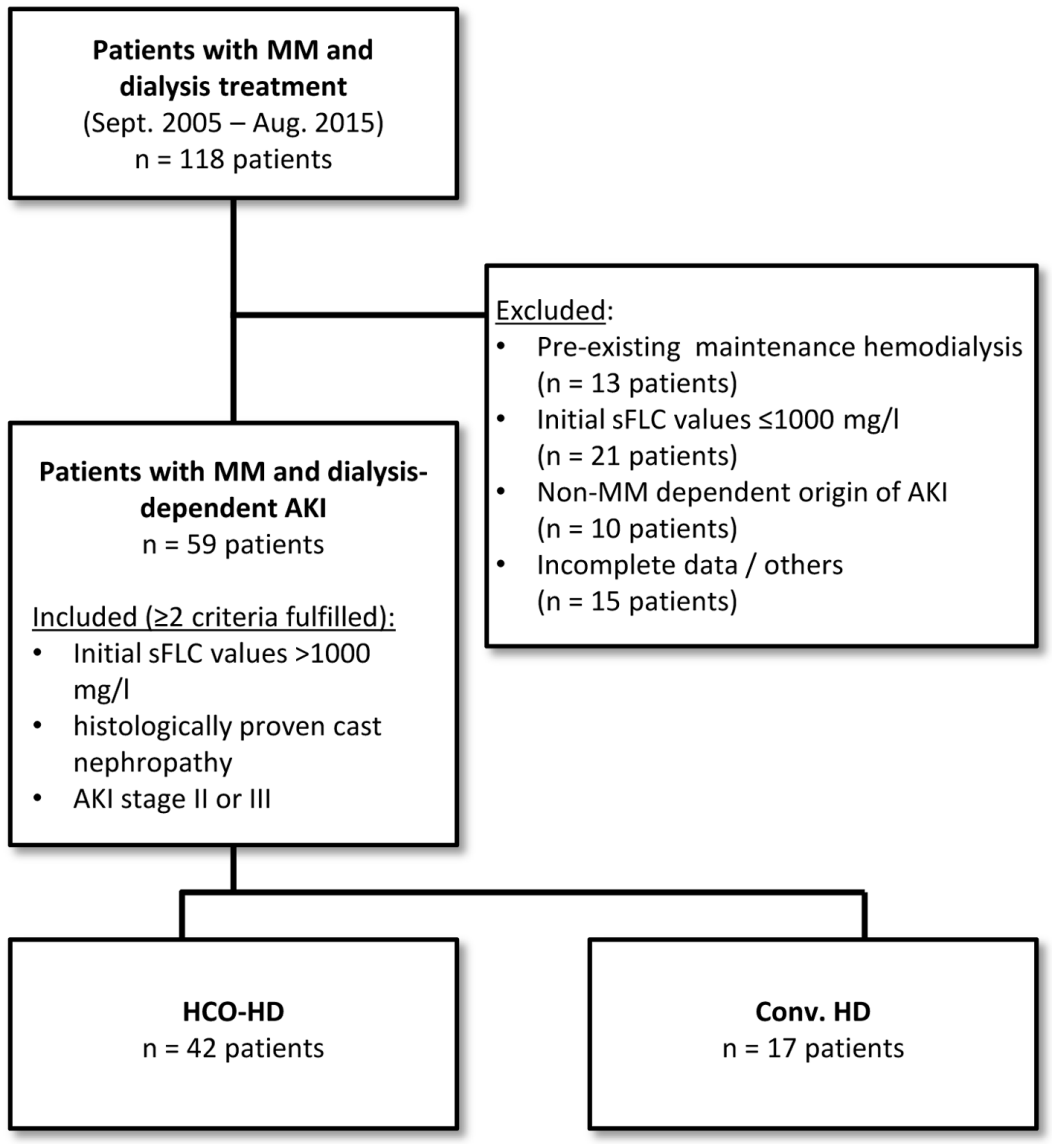
Flow chart displaying the patient selection. Abbrev.: MM—Multiple Myeloma; AKI—acute kidney injury; sFLC—serum free light chain; HCO-HD—High cut-off dialysis; conv. HD—conventional hemodialysis

Patients consecutively admitted to our hospital between September 2005 (implementation of the Freelite^®^ Assay at our center to measure the serum concentration of kappa and lambda sFLC) and August 2015 with MM and dialysis-dependent AKI were retrospectively identified by ICD-10 GM (C90.0-) and OPS codes for dialysis procedures (Fig 1). Hemodialysis was initiated for clinical reasons according to the current Kidney Disease Improving Global Outcomes (KDIGO) criteria.[13] Patients already receiving maintenance hemodialysis were excluded. Two of the following selection criteria were mandatory for inclusion in analysis: a) histologically proven cast nephropathy, b) high sFLC values (>1000 mg/l) and c) AKI stage II or III at the time of hospital admission.

Due to a lack of recommendations in current guidelines, decision for the use of HCO-HD or conv. HD was the result of an individual case-discussion between the consultant haematologist and the consultant nephrologist. Therefore criteria for selection of the extracorporeal treatment mode were not standardized but mainly based on physician’s opinion. In addition to extracorporeal treatment, in-house chemotherapy was always administered.

Clinical and laboratory data were obtained from the electronic medical records. The diagnosis of MM was confirmed and included bone marrow biopsy according to the World Health Organization (WHO) criteria.[14] Staging of MM was performed according to the International Staging System (ISS).[15] All patients gave written informed consent prior to the initiation of any medical treatment. Approval for this investigation was obtained from the Ethics Board of the Westfalian Wilhelms-University Muenster and the Physicians Chamber of Westfalia-Lippe, Germany (Permit Number: 2015-696-f-S).

AKI at initiation of dialysis was classified post hoc by means of the current KDIGO criteria.[16] Urine output data were not available and therefore not included in our AKI definition. Pre-existing chronic kidney disease (CKD) was defined as a documented estimated glomerular filtration rate (eGFR) <60 ml/min/1.73 m^2^ >6 months prior to admission. eGFR was calculated using the CKD-EPI (Chronic Kidney Disease Epidemiology Collaboration) creatinine equation.[17] sFLC were measured by the Freelite^®^ immunoassay (The Binding Site, Birmingham, Great Britain) in our hospital laboratory.

### Extracorporeal treatment

Renal replacement therapy was performed almost daily (at least five procedures per week) in the HCO-HD group or at least three times per week in the conv. HD group. The corresponding average treatment times were 360 and 240 minutes, respectively. For conv. HD, common high-flux class filters (FX series, Fresenius Medical Care GmbH, Bad Homburg, Germany) were generally used. HCO-HD was performed using HCO-1100^®^ or Theralite^®^ dialyzers (both Gambro GmbH, Hechingen, Germany). In February 2012, delivery of HCO-1100^®^ dialyzers was discontinued by Gambro and replaced by Theralite^®^ filters. Blood flow rates were 250–350 ml per min, and dialysate flow rates were approximately 500 ml per min for both, conventional and HCO filters. If not contraindicated, heparin was used for anticoagulation. HCO-HD was usually discontinued as soon as sFLC concentrations were <1000 mg/l, independent of renal function. In case of decrease in sFLC <1000 mg/l but persistent need for hemodialysis, HCO-HD treatment was replaced by dialysis with common high-flux class filters. Conv. HD was discontinued once patients regained adequate urine output and eGFR values were >15 ml/min/1.73 m^2^.

### Outcome

Renal recovery, the primary outcome, was defined as independence from dialysis before death, combined with stable kidney function with an eGFR >15 ml/min/1.73 m^2^ at a minimum of two weeks after the last dialysis treatment. Data were censored at the time of death or at day 90 of follow-up. A sustained sFLC response, the secondary outcome, was defined as a decrease in sFLC values below a cut-off of 1000 mg/l within 30 days and lasting up to day 90 after the start of extracorporeal treatment. Overall survival was calculated from the first day of dialysis to death from any cause. Patients who did not suffer from any event within the follow-up period were censored after 1 year. Response was classified according to the criteria of the International Myeloma Working Group.[18]

### Statistical analysis

Data are presented as absolute numbers, percentages, and medians with corresponding 25th and 75th percentiles (IQR). Differences between conv. HD and HCO-HD were analyzed by the two-sided Mann-Whitney U test in the case of continuous variables. Chi-squared analysis was used to compare categorical variables. The distribution of the time-to-event variables was estimated using the Kaplan-Meier method with log-rank testing.

Parameters associated with renal recovery were identified by univariate and multivariate logistic regression models. Selection of variables was performed *a priori* by determining probable confounders based on differences in baseline characteristics between patients with different dialysis modalities and based on theoretical considerations.

Variables found to be statistically significant at a 10% level in the univariate analysis were included in the multivariate model. In an explorative approach, all variables incorporated in the multivariate logistic regression model were concurrently subjected to a decision tree analysis (exhaustive chi-squared automatic interaction detection, CHAID, split alpha = 0.05, no correction for multiple testing) to recursively identify the best predictors and patient subgroups with different prognoses for renal recovery. Decision tree growth was stopped at node splits resulting in subgroups smaller than 10 patients.

The time to sFLC reduction below 1000 mg/l was estimated by fitting non-linear regression models according to subgroup (HCO HD, conv. HD, renal recovery, no renal recovery) to the logarithmized sFLC data. The underlying model was as follows: log10 (sFLC) = intercept * time[days]k. The data were fitted using PROC NLIN in the SAS software. The resulting model coefficients (intercept, k) are summarized in S1 Table.

Two-sided p-values <0.05 were considered statistically significant. Statistical analyses were performed using IBM SPSS Statistics for Windows, version 22.0 (IBM Corp., Armonk, NY, USA); GraphPad Prism 5 for Windows, version 5.01 (GraphPad Software, La Jolla, CA, USA); and SAS/STAT 13.2 software (version 9.4 of the SAS system for Windows, SAS Institute Inc., Cary, NC, USA).

## Results

The final dataset consisted of 59 patients with MM and dialysis-dependent AKI at the time of hospital admission. The detailed flow chart depicting the patient selection process is displayed in Fig 1. The patients’ demographics, clinical and laboratory baseline characteristics, and treatment details are shown in Table 1. Thirty-two patients (54%) presented with MM as an initial diagnosis, and 27 patients (46%) had relapsed MM or refractory disease. The majority of patients presented with MM in ISS stage III (96.6%), and the renal status at admission was predominantly AKI III (71%). Almost all patients who underwent renal biopsy had cast nephropathy. A total of 17 patients were treated with conv. HD, whereas 42 patients received HCO-HD for extracorporeal sFLC elimination. The two groups showed no significant differences in patient demographics and baseline disease characteristics. However, 76.2% of the patients in the HCO-HD group were treated with novel agents (bortezomib, thalidomide, or lenalidomide), compared with only 23.5% of the patients in the conv. HD group. In addition, there were small but significant differences in both treatment groups regarding the time period from hospital admission to initiation of chemotherapy or extracorporeal treatment (Table 1). In the HCO-HD group, chemotherapy and extracorporeal treatment were initiated nearly simultaneously. In the conv. HD group, dialysis was started in median four days before chemotherapy initiation.

**Table 1:** Patient characteristics.

		*Type of extracorporeal treatment*		
	All patients (n = 59)	HCO-HD (n = 42)	Conv. HD (n = 17)	*P*-value^a^
**Age**	63.1 (56.3–69.2)	64.4 (56.7–69.8)	58.4 (53.7–66.4)	0.132
>60 years	31 (52.5)	25 (59.5)	6 (35.3)	0.090
**Gender, male/female**	30/29 (50.8/49.2)	20/22 (47.6/52.4)	10/7 (58.8/41.2)	0.435
**Status**				
Primary diagnosis	32 (54.2)	22 (52.4)	10 (58.8)	0.652
Relapse/refractory	27 (45.8)	20 (47.6)	7 (41.2)	
**sFLC type**				
Kappa	36 (61)	26 (61.9)	10 (58.8)	0.826
lambda	23 (39)	16 (338.1)	7 (41.2)	
kappa [mg/l]	8450 (3170–16100)	8545 (3500–16600)	5130 (1955–19650)	0.462
lambda [mg/l]	6100 (3310–12400)	5250 (3037–9677)	12400 (3310–15900)	0.109
**MM type**				
IgG kappa	15 (25.4)	12 (28.6)	3 (17.6)	0.352
IgG lambda	10 (16.9)	8 (19.0)	2 (11.8)	
IgA kappa	3 (5.1)	3 (7.1)	-	
IgA lambda	3 (5.1)	1 (2.4)	2 (11.8)	
sFLC kappa only	19 (32.2)	12 (28.6)	7 (41.2)	
sFLC lambda only	9 (15.3)	6 (14.3)	3 (17.6)	
**ISS stage**				
I	1 (1.7)	1 (2.4)	-	0.500
II	1 (1.7)	1 (2.4)	-	
III	57 (96.6)	40 (95.2)	17 (100)	
**Previous renal status**				
pre-existing CKD (≥3) prior to MM	16 (27.1)	10 (23.8)	6 (35.3)	0.376
previous serum creatinine [mg/dl]	1.0 (0.8–1.2)	1.0 (0.8–1.1)	1.0 (0.8–1.6)	0.489
previous eGFR [CKD-EPI] [ml/min]	74.5 (50.9–80.7)	75.6 (61.7–80.9	73.7 (42.5–79.3)	0.384
**Renal status at presentation**				
Serum creatinine [mg/dl]	4.5 (2.9–6.9)	4.4 (2.8–6.1)	5.0 (3.8–7.8)	0.315
BUN [mg/dl]	63 (44–81)	60.5 (39.0–75.5)	76 (51–122)	0.064
AKI [KDIGO]				
AKI I	7 (11.9)	4 (9.5)	3 (17.6)	0.679
AKI II	10 (16.9)	7 (16.7)	3 (17.6)	
AKI III	42 (71.2)	31 (73.8)	11 (64.7)	
**Renal biopsy**				
Cast Nephropathy	16 (27.1)	14 (33.3)	2 (11.8)	0.214
Others	1 (1.7)	-	1 (5.9)	
Declined or contraindicated	42 (71.2)	28 (66.7)	14 (82.4)	
**Laboratory data**				
Haemoglobin [g/dl]	9.1 (8.3–10.2)	9.0 (8.3–10.3)	9.2 (8.0–9.8)	0.782
Platelet counts [x10^9^/l]	160 (83–216)	158 (86–216)	175 (74–217)	0.770
LDH [U/l]	278 (209–427)	285 (219–492)	272 (198–378)	0.407
Uric acid [mg/dl]	8.4 (6.2–10.2)	8.6 (6.8–10.3)	8.0 (5.7–10.2)	0.574
Beta2-Microglobulin [mg/l]	13.25 (7–31.5)	11.3 (7–17.6)	26.4 (8.5–36.7)	0.124
**Chemotherapy regimen**				
Days from hospital admission to chemotherapy initiation	2 (0–6)	2 (0–6)	4 (2–11)	0.033
Protocol	36 (61)	32 (76.2)	4 (23.5)	<0.0001^b^
Novel agents (bortezomib, thalidomide, lenalidomide)				
Cyclophosphamide-based	10 (17)	8 (19)	2 (11.8)	
Dexamethasone	5 (8.5)	1 (2.4)	4 (23.5)	
Anthracycline-based	5 (8.5)	-	5 (29.4)	
Melphalan/Bendamustin-based	3 (5.1)	1 (2.4)	2 11.8)	
**Extracorporeal treatment**				
Days from hospital admission to first treatment	1 (0–3)	2 (1–3)	0 (0–2.5)	0.004
Treatment period (days)^c^	14 (7–24)	10.5 (6.7–22.2)	19 (6–40.5)	0.215
number of treatment sessions (n)	8 (5–16)	8 (5–14.5)	9 (3.5–18.5)	0.782

Median and interquartile range reported for continuous variables and frequency with percentage reported for categorial variables.

^a^ Comparison of HCO versus conventional haemodialysis. Continuous variables are compared with Mann-Whitney-Test. Categorial are compared with Chi-Square-test.

^b^ Protocols including novel agents versus other combinations

^c^ time period between the first and the last extracorporeal treatment after hospital admission

Abbrev.: HCO-HD—High cut-off dialysis; conv. HD—conventional haemodialysis; sFLC—serum free light chain; MM—Multiple Myeloma; ISS—International staging system; CKD—chronic kidney disease; eGFR—estimated glomerular filtration rate; CKD-EPI—Chronic Kidney Disease Epidemiology Collaboration; BUN—Blood Urea Nitrogen; KDIGO—Kidney Disease: Improving Global Outcome; AKI—acute kidney injury; LDH—Lactate dehydrogenase

### Outcomes

**Fig 2:**
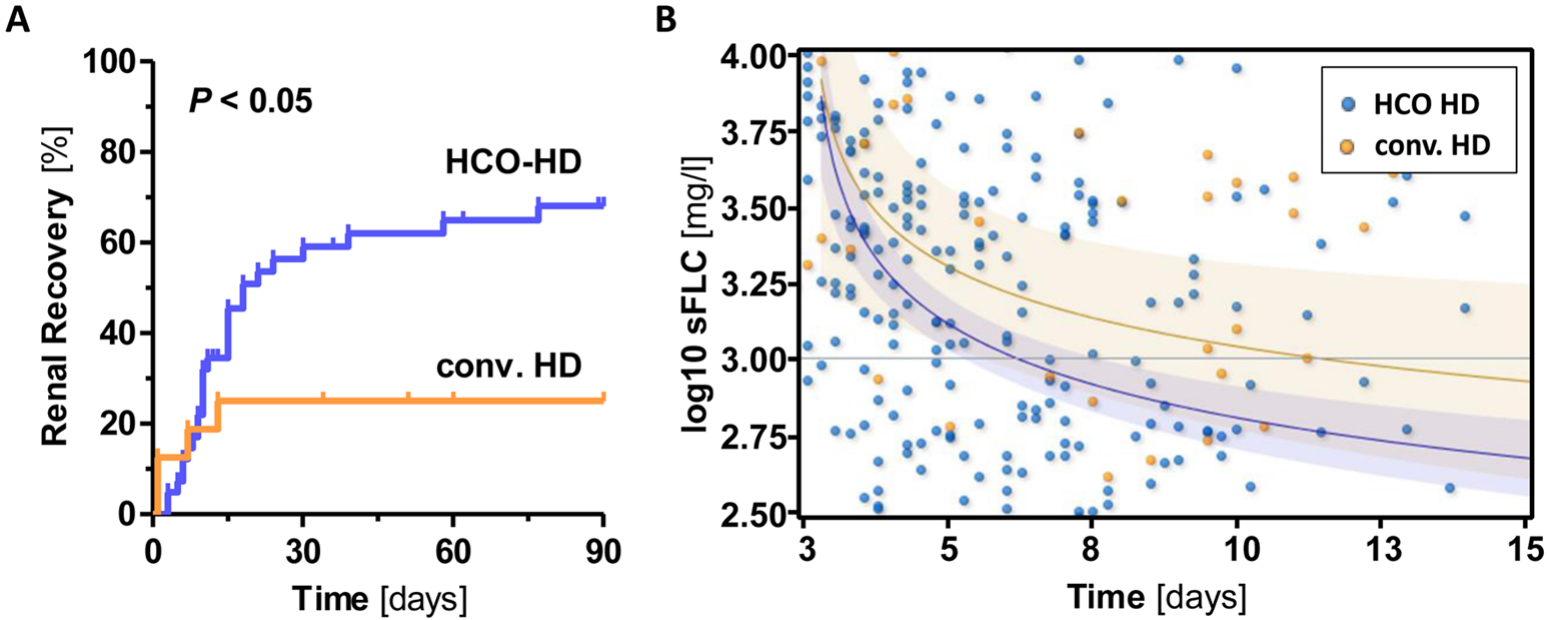
Renal recovery rate and regression curves of sFLC values in HCO-HD and conventional HD. A) Renal recovery according to the type of extracorporeal treatment. A total of 64.3% (27 of 42 patients) of patients in the HCO-HD group achieved freedom from dialysis within 90 days, compared with 29.4% (5 of 17 patients) in the conventional HD group (p = 0.014). B) Corresponding non-linear regression curves (including 95% confidence bands) of sFLC values in both subgroups. On average, patients receiving HCO-HD experienced a therapeutic decrease in sFLC values <1000 mg/l (reference line) on day 14.5, whereas this decrease did not occur until day 36 in the conv. HD group.

The HCO-HD group showed a significantly higher rate of renal recovery compared with the conv. HD group (64.3% vs. 29.4%, odds ratio [OR] 4.3, 95% confidence interval [CI] 1.3–14.6, p = 0.014), as shown by the Kaplan-Meier curves (Fig 2A). Furthermore, a sustained sFLC response was observed more often in HCO-HD patients (83.3%) compared with conv. HD patients (29.4%) (p = 0.007) (Table 2). In addition, there was no significant difference in renal recovery with regard to which HCO filter was used (HCO-1100 *vs*. Theralite; data not shown).

**Table 2:** Renal response and clinical outcome.

		*Type of extracorporeal treatment*		
	All patients (n = 59)	HCO-HD (n = 42)	Conv. HD (n = 17)	*P*-value^a^
**Renal recovery at day 90**^b^	32 (54.2)	27 (64.3)	5 (29.4)	0.014
Serum creatinine at day 90	1.1 (0.8–1.9)	1.4 (0.9–1.9)	0.8 (0.6–1.0)	0.022
eGFR [CKD-EPI] at day 90 [ml/min]	13 (40.6)	9 (33.3)	4 (80)	0.128
≥60				
30–59	10 (31.3)	9 (33.3)	1 (20)	
15–29	6 (18.8)	6 (22.2)	-	
<15 (without dialysis)	-	-	-	
Missing data	3 (9.4)	3 (11.1)	-	
**Sustained sFLC response**^c^	30 (50.8)	26 (61.9)	4 (23.5)	0.007
**Mortality**				
day 90	12 (20.3)	9 (21.4)	3 (17.6)	0.741
one year	22 (37.3)	13 (31.0)	9 (52.9)	0.117
**Myeloma response (IMWG)**				
sCR/CR	5 (8.5)	5 (11.9)	-	0.224
VGPR	8 (13.6)	7 (16.7)	1 (5.9)	
PR	9 (15.3)	8 (19)	1 (5.9)	
SD	6 (10.2)	3 (7.1)	3 (17.6)	
PD	15 (25.4)	12 (28.9)	3 (17.6)	

Median and interquartile range reported for continuous variables and frequency with percentage reported for categorial variables.

^a^ Comparison of HCO versus conventional haemodialysis. Continuous variables are compared with Mann-Whitney-Test. Categorial are compared with Chi-Square-test.

^b^ death-censored

^c^ Defined as drop of sFLC values below a cut-off of 1000 mg/dl within 30 days after start of extracorporeal treatment.

Abbrev.: HCO-HD—High cut-off dialysis; conv. HD—conventional haemodialysis; eGFR—estimated glomerular filtration rate; CKD-EPI—Chronic Kidney Disease Epidemiology Collaboration; sFLC—serum free light chain; sCR—stringent Complete Remission; CR—Complete Remission; VGPR—Very Good Partial Remission; PR—Partial Remission; SD—Stable Disease; PD—Progressive Disease

The average duration of sFLC required to reach values <1000 mg/l was 14.5 days in the HCO-HD group, compared with 36 days in the conv. HD group. The corresponding regression curves are shown in Fig 2B. Of course, we cannot exclude an additional effect of the varying antimyeloma therapy on this parameter. Regardless, a sustained decline of sFLC values to <1000 mg/l was associated with a higher rate of renal recovery (S1 Fig). Specifically, patients with a sustained sFLC reduction had a renal recovery rate of 70% (21 of 30 patients), compared with a rate of 37.9% (11 of 29 patients) among patients with sFLC values ≥1000 mg/dl (OR = 3.8, 95%CI 1.3–11.2, p = 0.015). The associated regression curve of sFLC is shown in S1 Fig. Predictors of a sustained sFLC decrease are presented in S2 Table.

The corresponding one-year overall survival of patients with renal recovery was 78.1%, compared with 44.4% of dialysis-dependent patients (p = 0.117; data not shown). Among the survivors free of dialysis, the median eGFR (CKD-EPI) at day 90 was lower in the HCO-HD group than in the conv. HD group (48 and 100 ml/min/1.73 m^2^, respectively, p = 0.035). Nevertheless, the corresponding one-year survival rates showed a trend toward being higher in the HCO-HD group (69%) than in the conv. HD group (47.1%) (p = 0.117).

Regardless of the chemotherapeutical regimen, HCO-HD improved renal recovery rate compared to conv. HD. Patients treated with novel agents revealed a renal recovery rate of 50.0% (conv. HD) which could be increased to 65.6% by HCO-HD. If treated with protocols excluding any novel chemotherapeutic agent the corresponding rates of renal recovery were 23.1% (conv. HD) compared to 60.0% (HCO-HD), respectively. Thus type of chemotherapy itself showed a trend towards a higher rate of renal recovery for the use of novel agents in univariate analysis (Table 3, p = 0.066).

**Table 3:** Univariate and multivariate analysis for factors associated with renal recovery.

	*Univariate*		*Multivariate*	
	OR (95% CI)	*P*-value	OR (95% CI)	*P*-value
**Primary diagnosis *vs*. Relapse/refractory**	0.91 (0.32–2.54)	0.852		
**Male gender**	0.40 (0.14–1.15)	0.090	0.46 (0.13–1.62)	0.226
**Age**	0.99 (0.94–1.05)	0.861		
**Previous eGFR [ml/min; CKD-EPI]**	1.00 (0.98–1.02)	0.740		
**Serum Creatinine**^a^ **[mg/dl]**	1.27 (1.01–1.58)	0.038	1.14 (0.86–1.51)	0.369
**BUN**^a^ **[mg/dl]**	1.03 (1.01–1.05)	0.010		
**AKI III**^a^ **[KDIGO] *vs*. others**	1.08 (0.35–3.33)	0.899		
**sFLC**^a^ **[mg/dl]**	1.00 (1.00–1.00)	0.410		
**Haemoglobin**^a^ **[g/dl]**	0.84 (0.59–1.20)	0.341		
**Platelet count**^a^ **[x10**^**9**^**/l]**	1.00 (0.99–1.00)	0.536		
**LDH**^a^ **[U/l]**	1.00 (1.00–1.00)	0.434		
**Albumin**^a^ **[g/dl]**	0.98 (0.91–1.05)	0.570		
**Calcium**^a^ **[mmol/l]**	0.96 (0.45–2.06)	0.916		
**Uric acid**^a^ **[mg/dl]**	1.22 (0.97–1.52)	0.089	1.30 (1.01–1.70)	**0.045**
**Novel agents *vs*. others**	2.75 (0.94–8.09)	0.066	2.17 (0.58–8.18)	0.249
**HCO-HD *vs*. conv. HD**	4.32 (1.28–14.62)	0.019	6.08 (1.51–24.55)	**0.011**

^a^ Laboratory data at presentation.

Abbrev.: CKD—Chronic Kidney Disease; eGFR—estimated glomerular filtration rate; CKD-EPI—Chronic Kidney Disease Epidemiology Collaboration; BUN—Blood Urea Nitrogen; AKI—acute kidney injury; KDIGO—Kidney Disease: Improving Global Outcome; sFLC—serum free light chain; LDH—Lactate dehydrogenase; HCO-HD—High cut-off dialysis; conv. HD—conventional haemodialysis

### Predictors of renal recovery

Among the clinical baseline parameters, only serum creatinine and blood urea nitrogen were associated with dialysis independence in the univariate analysis (p = 0.038 and p = 0.010, respectively). In the multivariate regression analysis, uric acid (p = 0.045) and HCO-HD (p = 0.011) were identified as independent predictors of renal recovery (Table 3).

**Fig 3:**
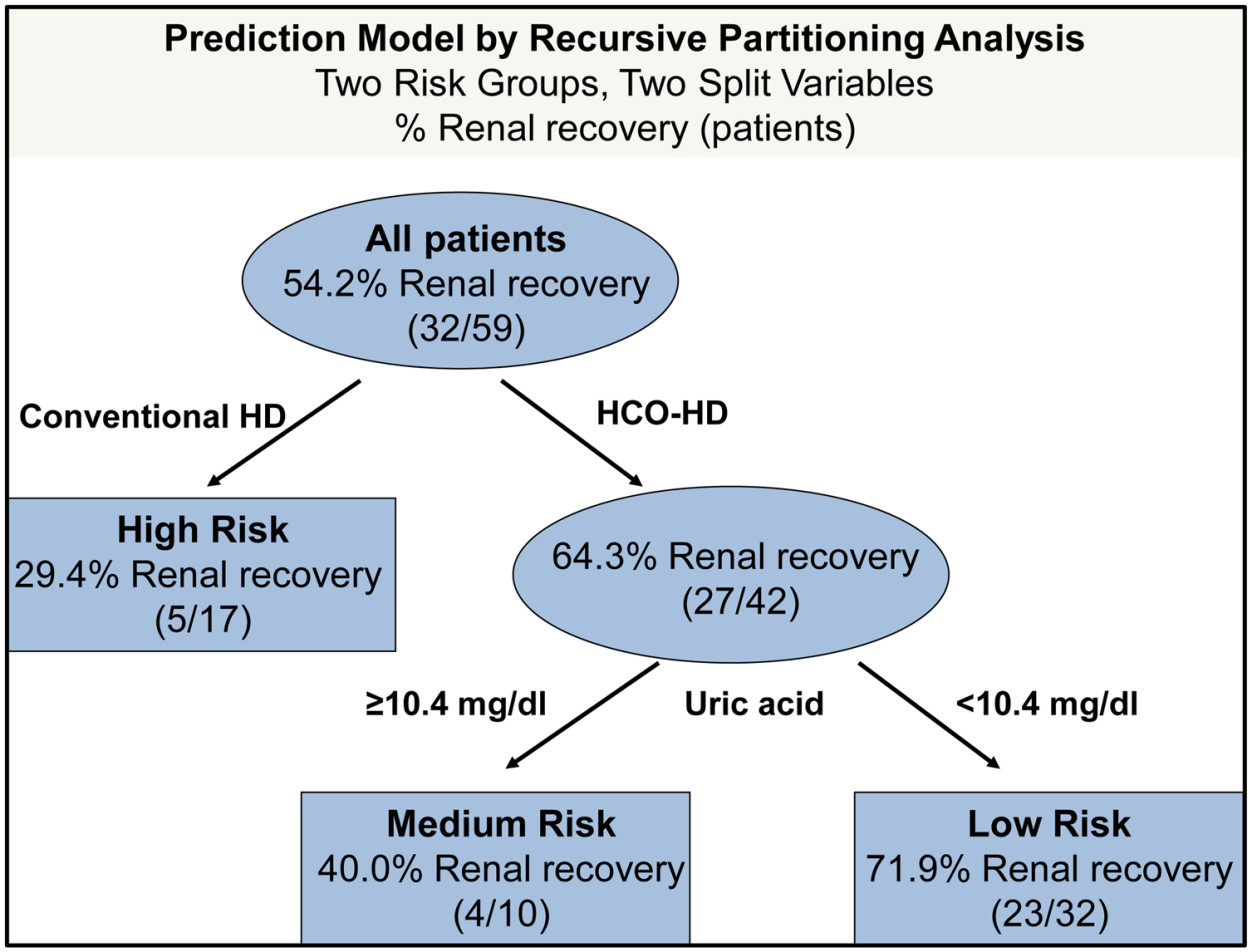
Prediction model of renal recovery by recursive partitioning analysis. Prediction model developed by recursive partitioning analysis to estimate the risk class of renal recovery based on two split variables: mode of extracorporeal therapy (HCO-HD *vs*. conv. HD) and serum uric acid values (<10.4 mg/dl *vs*. ≥10.4 mg/dl) before therapy initiation. With the application of HCO therapy, the rate of renal recovery doubled from 29% to 64% of patients. Further assessment of uric acid values predicts the probability of renal recovery more precisely, resulting in a medium-risk class (40.0% renal recovery) or low-risk class (71.9% renal recovery).

Finally, all variables incorporated into the multivariate model were concurrently subjected to decision tree analysis (recursive partitioning). Using this approach, we recursively identified a sequence of two split variables (HCO-HD *vs*. conv. HD and uric acid <10.4 mg/dl) that best separated patient subgroups according to prognosis of renal recovery (low *vs*. medium *vs*. high risk group) (Fig 3). In both HD groups, high initial uric acid values were associated with low renal recovery rates. The corresponding Kaplan-Meier curves of renal recovery stratified according to risk class are shown in S2 Fig.

## Discussion

To the best of our knowledge, this is the first case-control study comparing HCO-HD with conv. HD for dialysis-dependent AKI secondary to MM. Our results are in line with results from previous studies indicating that a rapid and early reduction in sFLC is predictive of renal recovery. The rate of renal recovery in the HCO group (64%) was comparable to that in previous reports.[10–12,19,20] Furthermore, we found that HCO-HD was independently associated with a greater sFLC decline and a higher rate of renal recovery than conv. HD, as shown by two different statistical multivariate approaches.

Almost a decade ago, Hutchison and coworkers discovered that protein-leaking dialysis filters (HCO filters) allowed the removal of immunoglobulin sFLC in large amounts.[9] Subsequent studies utilized HCO filters and found that renal recovery in the myeloma kidney depends on the early reduction of sFLC and is associated with a significant survival advantage.[10–12] In these and other studies, the rate of renal recovery from dialysis-dependent AKI secondary to MM was consistently >60%.[10–12,19,20] Importantly, the large majority of patients with renal function recovery after combination therapy with chemotherapy and HCO-HD did not require further dialysis during long-term follow-up.[21] Thus, early reduction of sFLC is currently broadly accepted as a major therapeutic goal in dialysis-dependent AKI secondary to MM. However, because none of the published studies included a control group of patients treated with conv. HD (i.e., without extracorporeal sFLC removal), the additional benefit of HCO-HD as an add-on treatment to standard chemotherapy alone has not been addressed to date. Two randomized controlled trials, EULITE (European Trial of Free Light Chain Removal by Extended Haemodialysis in Cast Nephropathy) and MYRE (Studies in Patients with Multiple Myeloma and Renal Failure Due to Myeloma Cast Nephropathy), are ongoing. However, owing to the rare incidence of dialysis-dependent MM, patient recruitment is protracted.[22,23] To date, only one prospective observational case series comparing HCO-HD with conv. HD for AKI secondary to MM has been published. Peters et al. found that 3 of 5 HCO-HD-treated patients, but none of the 5 patients treated with conv. HD, recovered from dialysis.[24]

In our study we observed a trend in the corresponding one-year survival rates toward a higher survival in the HCO-HD group (69% *vs*. 47.1% in the conv. HD group, p = 0.117). This is in line with previous reports on HCO-HD indicating an improved long-term outcome of MM patients with recovery from dialysis-dependency.[21] Interestingly, eGFR at day 90 in patients recovering independent renal function was higher in the conv. HD group (100 ml/min/1.73m^2^ *vs*. 48 ml/min/1.73 m^2^ in the HCO-HD group). As HCO-HD may have a greater ability to prevent dialysis dependence, only a small number of five conv. HD patients were alive at day 90 and were compared to a considerably greater number of HCO-HD patients (n = 27). It is intriguing to speculate that some patients with lower eGFR values in the HCO-HD group might have been remained on permanent HD if they had been treated with conv. HD instead.

Interestingly, our data show no differences in outcomes according to disease status (*de novo vs*. relapsed MM). In addition, novel agents only trended toward yielding a better renal recovery rate in the univariate analysis. Given the retrospective nature of our study, we cannot exclude the possibility that the adoption of novel chemotherapy protocols, including bortezomib, thalidomide, and lenalidomide, during the long study period may have influenced the results. Approximately fifty percent of MM patients with AKI who respond to bortezomib-based regimens will have a resulting significant improvement in renal function within a few weeks after therapy initiation.[1,2,25] It is possible that the findings did not reach statistical significance because of the limited number of patients treated without novel agents. This, however, would further emphasize the importance of HCO-HD for renal recovery.

As novel chemotherapy agents have become the standard of care, it is important to estimate a possible therapeutic benefit of HCO-HD prior to therapy initiation. Decision tree analysis not only identified HCO-HD as a paramount variable associated with a favorable renal outcome but also introduced uric acid as a further marker that separated patients in the HCO-HD group into subgroups with low (renal recovery rate 72%) or medium (renal recovery rate 40%) risk. Elevated uric acid has already been described as a risk factor for AKI in various clinical scenarios, such as rhabdomyolysis,[26] severe burn injury,[27] cardiovascular surgery[28] and hospitalized patients in general.[28] In line with these studies, we noted that uric acid values were associated with renal outcome independent of the mode of extracorporeal therapy (S2 Fig). Elevated uric acid in MM may reflect a combination of impaired renal function, disease severity, and sub-clinical tumor-lysis syndrome. Although interesting, this finding is hypothesis-generating in nature and requires validation in future studies.

Some limitations deserve discussion. First, this single-center study was retrospective and included only a limited number of patients. Second, the assignment of the patients into one of the groups was not random but was influenced by physicians’ decisions reflecting various parameters, including the availability of HCO-HD. This approach, in contrast to prospective randomization, carries the limitation of introducing unknown confounding variables that influence the outcome. In the course of time, further development of treatment standards influenced myeloma outcome and also the results of this study. However, our data show a beneficial effect of HCO-HD on renal recovery independently of chemotherapy regimen. In particular, a more frequent determination of sFLC in the HCO-HD group to guide the duration of extracorporeal treatment may have caused a steeper sFLC decrease. To compensate for this phenomenon, we calculated regression curves and used a wide range (i.e., 30 days) for the definition of a sustained sFLC response. Third, renal biopsy was not performed for clinical reasons in ~3/4 of the cases. However, the prevalence of cast nephropathy among patients who underwent biopsy was >90%. It is reasonable to assume that the majority of patients had AKI due to cast nephropathy. Fourth, we cannot ignore the fact that the threshold for initiating HCO-HD in AKI secondary to MM is much lower than that for initiating conv. HD. In fact, we were surprised to observe quite similar (acute and chronic) renal parameters in the two treatment groups. Finally, due to the retrospective nature of the study, data on urinary output could not be retrieved. Although the data from this study certainly do not exceed level III evidence (according to Oxford (UK) CEBM Levels of Evidence), they enable the generation of a hypothesis, and further prospective trials, including those currently in the recruitment phase, are warranted to unequivocally demonstrate the superiority of HCO-HD.

In summary, results from this retrospective case-control study suggest an additional benefit of HCO-HD in sFLC removal and renal outcome in dialysis-dependent AKI secondary to MM. Add-on therapy with HCO-HD should be considered for all patients suffering high sFLC values.

## Supporting Information

S1 Fig Correlation of renal recovery and sFLC value. A) Renal recovery in patients with and without a sustained reduction of sFLC values (cut-off <1000 mg/l) within 30 days after therapy initiation. A total of 70% (21 of 30 patients) of patients with a rapid fall in sFLC values achieved freedom from dialyses, whereas 62.1% of the patients without a sFLC reduction (18 of 29) remained dialysis-dependent (p = 0.028). B) Calculated non-linear regression curves (including 95% confidence bands) of sFLC values in patients with (32 patients) and without (27 patients) renal recovery within 90 days after therapy initiation. Patients with successful renal recovery experienced a decrease in sFLC values to <1000 mg/l an average of 9 days after therapy initiation. The average time to achieve sFLC values <1000 mg/l in patients without renal recovery was greater than 90 days and could not be estimated from the data.
(PDF)

S2 Fig Correlation of renal recovery and baseline serum uric acid value. A) Relationship of baseline serum uric acid values with predicted probabilities of renal recovery in patients treated with HCO-HD (blue line and markers, n = 42 patients) and conv. HD (orange line and markers, n = 17 patients). The 95% confidence band of the predicted probability is shown. The level of baseline uric acid values determined the rate of renal recovery, independent of the extracorporeal treatment modality prior to therapy initiation. B) Renal recovery of patients based on three risk classes of the prediction model. Patients with a high risk of renal recovery received conv. HD (independent of uric acid values). Medium-risk patients presented with high uric acid values (≥10.4 mg/dl) and received HCO treatment. Only patients with low uric acid values and who received HCO-HD had a low risk for remaining dialysis-dependent.
(PDF)

S1 Table Fitting of sFLC data over time for non-linear regression analysis. (DOC)

S2 Table Univariate and multivariate analysis for factors associated with sustained sFLC decrease. (DOCX)
