# Supplementary figures and images for: Impact of High-Cut-Off Dialysis on Renal Recovery in Dialysis-Dependent Multiple Myeloma Patients: Results from a Case-Control Study
Source: PLoS One. 2016 May 6;11(5):e0154993. doi: 10.1371/journal.pone.0154993 (PMC4859546; doi:10.1371/journal.pone.0154993)

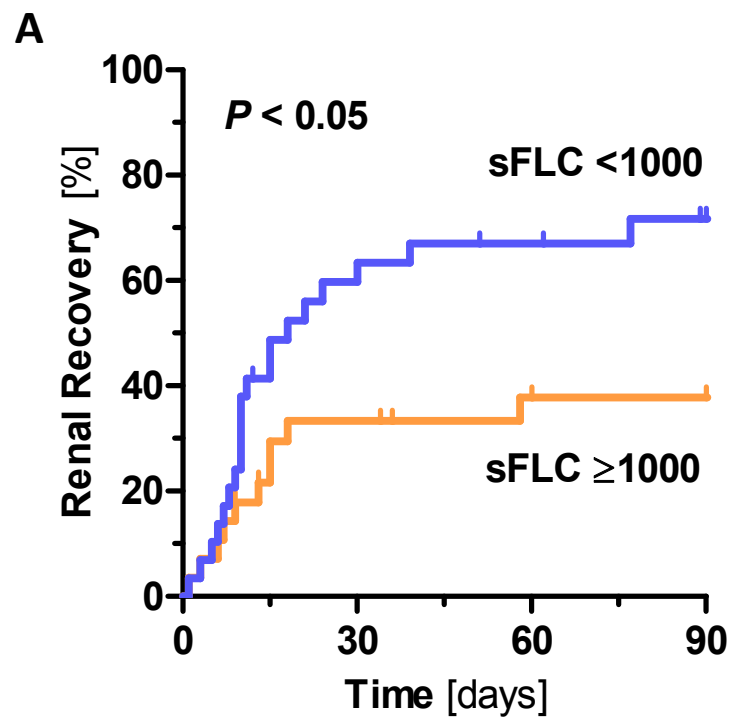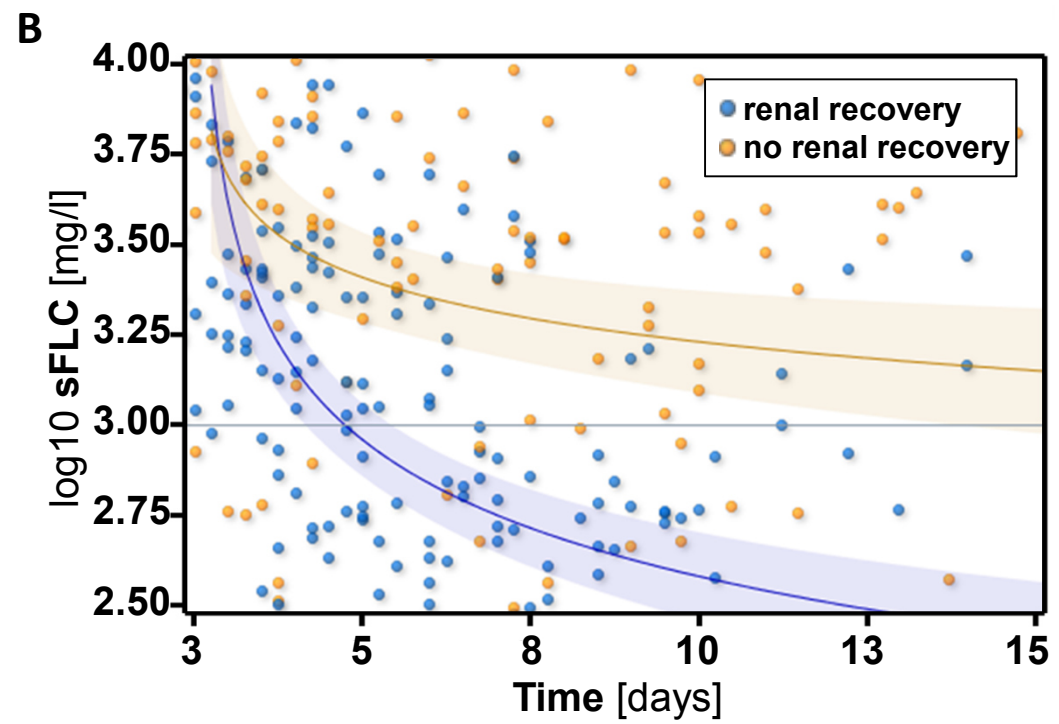

Supplement: S1 Fig — A) Renal recovery in patients with and without a sustained reduction of sFLC values (cut-off <1000 mg/l) within 30 days after therapy initiation. A total of 70% (21 of 30 patients) of patients with a rapid fall in sFLC values achieved freedom from dialyses, whereas 62.1% of the patients without a sFLC reduction (18 of 29) remained dialysis-dependent (p = 0.028). B) Calculated non-linear regression curves (including 95% confidence bands) of sFLC values in patients with (32 patients) and without (27 patients) renal recovery within 90 days after therapy initiation. Patients with successful renal recovery experienced a decrease in sFLC values to <1000 mg/l an average of 9 days after therapy initiation. The average time to achieve sFLC values <1000 mg/l in patients without renal recovery was greater than 90 days and could not be estimated from the data. (PDF) [file pone.0154993.s001.pdf]

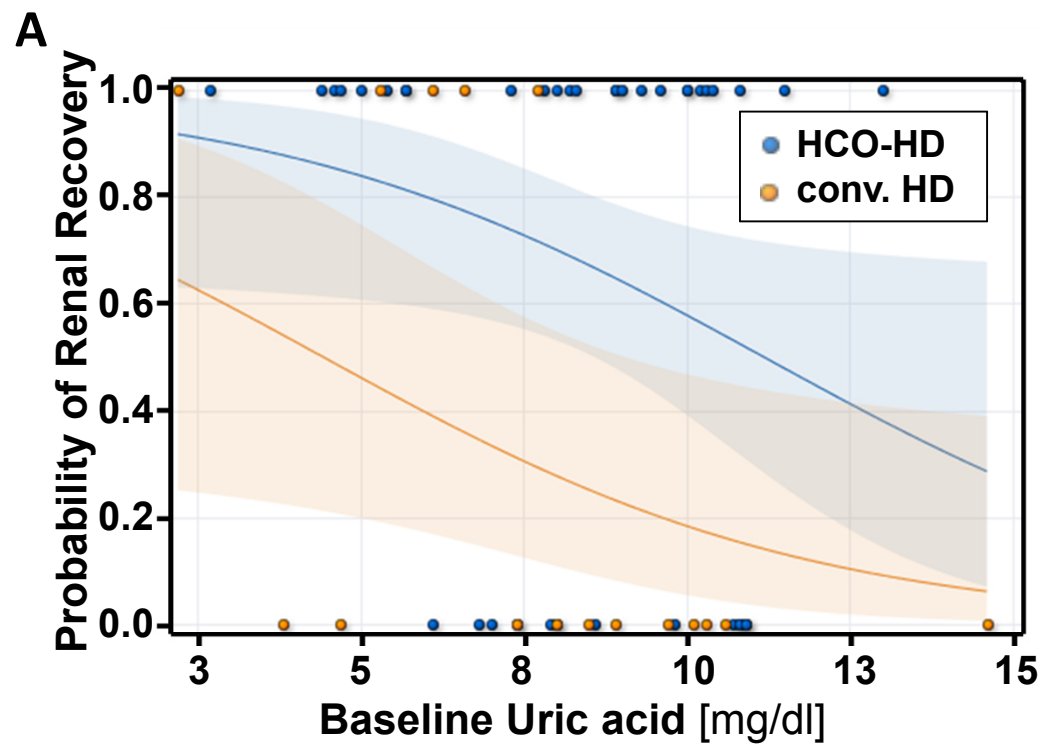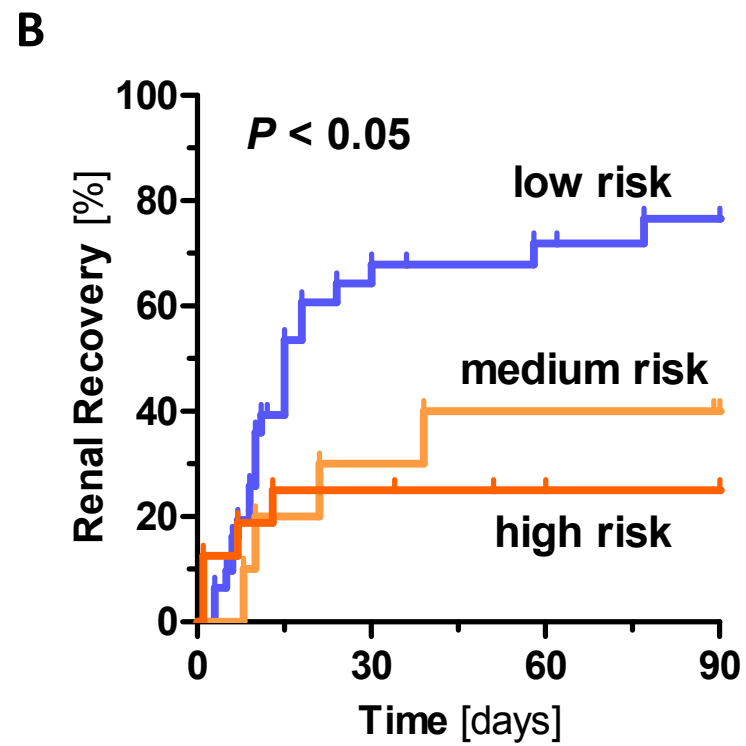

Supplement: S2 Fig — A) Relationship of baseline serum uric acid values with predicted probabilities of renal recovery in patients treated with HCO-HD (blue line and markers, n = 42 patients) and conv. HD (orange line and markers, n = 17 patients). The 95% confidence band of the predicted probability is shown. The level of baseline uric acid values determined the rate of renal recovery, independent of the extracorporeal treatment modality prior to therapy initiation. B) Renal recovery of patients based on three risk classes of the prediction model. Patients with a high risk of renal recovery received conv. HD (independent of uric acid values). Medium-risk patients presented with high uric acid values (≥10.4 mg/dl) and received HCO treatment. Only patients with low uric acid values and who received HCO-HD had a low risk for remaining dialysis-dependent. (PDF) [file pone.0154993.s002.pdf]
