# Supplementary material for: Impact of High-Cut-Off Dialysis on Renal Recovery in Dialysis-Dependent Multiple Myeloma Patients: Results from a Case-Control Study
Source: PLoS One. 2016 May 6;11(5):e0154993. doi: 10.1371/journal.pone.0154993 (PMC4859546; doi:10.1371/journal.pone.0154993)
Supplement: S1 Table — (DOC) [file pone.0154993.s003.doc]

**S1 Table. Fitting of sFLC data over time for non-linear regression analysis.**

|  | **HCO-HD (95%-CI)** | **Conv. HD (95%-CI)** |
| --- | --- | --- |
| Intercept | 3.8641 (3.5990 - 4.1292) | 3.9204 (3.2313 - 4.6096) |
| k | -0.0942 (-0.1201 - -0.0683) | -0.0748 (-0.1352 - -0.0144) |
|  | **Renal recovery (95%-CI)** | **No Renal Recovery (95%-CI)** |
| Intercept | 3.9418 (3.6126 - 4.2709) | 3.8156 (3.4768 - 4.1544) |
| k | -0.1244 (-0.1564 - -0.0923) | -0.0489 (-0.0803 - -0.0174) |

HCO-HD – High cut-off dialysis; conv. HD – conventional haemodialysis; CI – Confidence interval
