# Supplementary material for: Impact of High-Cut-Off Dialysis on Renal Recovery in Dialysis-Dependent Multiple Myeloma Patients: Results from a Case-Control Study
Source: PLoS One. 2016 May 6;11(5):e0154993. doi: 10.1371/journal.pone.0154993 (PMC4859546; doi:10.1371/journal.pone.0154993)
Supplement: S2 Table — (DOCX) [file pone.0154993.s004.docx]

**S2 Table. Univariate and multivariate analysis for factors associated with sustained sFLC decrease.**

|  | ***Univariate*** | |  | ***Multivariate*** | |  |
| --- | --- | --- | --- | --- | --- | --- |
|  | **OR (95% CI)** | ***P-*value** |  | **OR (95% CI)** | ***P-*value** |  |
| Primary diagnosis *vs.* Relapse/refractory | 1.41 (0.50 - 3.96) | 0.507 |  |  |  |  |
| Male gender | 1.07 (0.38 – 2.97) | 0.890 |  |  |  |  |
| Age | 0.99 (0.94 - 1.04) | 0.781 |  |  |  |  |
| Previous eGFR [CKD-EPI] | 1.56 (0.26 - 1.82) | 0.460 |  |  |  |  |
| Laboratory data at presentation:  Serum Creatinine [mg/dl] | 0.88 (0.72 – 1.08) | 0.249 |  |  |  |  |
| AKI III [KDIGO] *vs*. others | 0.96 (0.49 – 2.15) | 0.939 |  |  |  |  |
| sFLC [mg/dl] | 1.00 (1.00 - 1.00) | 0.777 |  |  |  |  |
| Haemoglobin [g/dl] | 1.23 (0.86 - 1.76) | 0.249 |  |  |  |  |
| Platelet count [x10^9^/l] | 1.00 (0.99 - 1.01) | 0.150 |  |  |  |  |
| LDH [U/l] | 1.00 (0.99 - 1.00) | 0.151 |  |  |  |  |
| Albumin [g/dl] | 0.90 (0.70 - 1.15) | 0.572 |  |  |  |  |
| Calcium [mmol/l] | 0.90 (0.75 - 1.08) | 0.266 |  |  |  |  |
| Uric acid [mg/dl] | 1.22 (0.97 – 1.52) | 0.089 |  |  |  |  |
| Novel agents *vs*. others | 3.03 (0.99 – 9.09) | 0.052 |  | 1.67 (0.48 – 5.81) | 0.417 |  |
| HCO-HD *vs.* conv. HD | 5.28 (1.46 – 19.03) | **0.011** |  | 5.29 (1.46 – 18.86) | **0.011** |  |

Abbr.: CKD - Chronic Kidney Disease; eGFR – estimated glomerular filtration rate; CKD-EPI - Chronic Kidney Disease Epidemiology Collaboration; AKI – acute kidney injury; KDIGO - Kidney Disease: Improving Global Outcome; sFLC – serum free light chain; LDH – Lactate dehydrogenase; HCO-HD – High cut off haemodialysis; conv. HD – conventional haemodialysis
